# Comparison of two respiratory function monitors for newborn mask ventilation: A randomised crossover study using simulation

**DOI:** 10.1016/j.resplu.2025.100937

**Published:** 2025-03-20

**Authors:** C.M. Ní Chathasaigh, A.E. Curley, E. O Currain

**Affiliations:** a Department of Neonatology, National Maternity Hospital, Dublin, Ireland; b School of Medicine, University College Dublin, Ireland

**Keywords:** Infant, Newborn, Resuscitation, Positive pressure ventilation, Face mask, Respiratory function monitor, Simulation

## Abstract

**Objectives:**

Respiratory function monitors (RFM) provide objective feedback on respiratory parameters during face mask ventilation. While traditional RFMs display detailed waveforms, newer devices use simplified, colour-coded graphics. We aimed to compare three RFM feedback methods against a no-feedback approach, assessing ventilation parameters and user interpretation.

**Methods:**

This simulation-based, crossover randomised study involved healthcare professionals at a tertiary neonatal centre, who received training on two RFMs: a “Coloured graphic” device (Monivent NeoTraining), offering monitor and sensor light feedback, and a “Flow curves” device (Respironics NM3). Participants performed positive pressure ventilation on a manikin across three phases: access to the “Coloured graphic” monitor and sensor light, “Light only”, and access to the “Flow curves” monitor, evaluated against a control phase with no feedback. An interpretation assessment followed. The primary outcome was the median difference in mask leak (%) between the control and the three intervention phases.

**Results:**

Data from 51 participants were analysed. Compared to the control, the median (IQR) mask leak (%) was significantly lower in the “Coloured graphic” phase (11% [7%–26%]; median difference: −13 [95% CI: −26 to −2]). No significant differences were observed in the “Light only” phase (22% [8%–39%]); median difference: −10 [95% CI: −25 to 5]), or “Flow curves” phase (44% [6%–73%]; median difference: 8 [95% CI: −2 to 18]). Although more participants correctly interpreted the “Coloured graphic” feedback, only a minority selected appropriate corrective actions.

**Conclusions:**

Objective feedback from the “Coloured graphic” RFM significantly reduced leak during mask ventilation.

## Background

Respiratory function monitors (RFM) provide real-time, objective feedback during face mask ventilation. While RFMs have been shown to improve mask ventilation performance in simulated environments,^1^, ^2^, ^3^ their impact on clinical outcomes during resuscitation has been inconsistent.^4^, ^5^, ^6^, ^7^ This may be attributed to infant movement, spontaneous breathing or cognitive overload from multiple monitors.^8^ RFMs display complex respiratory data in different formats, which may not prioritise ease of use and lead to difficulties in interpretation.^9^ While traditional devices display detailed waveforms (volume, pressure and flow curves), newer devices use simplified, colour-coded graphics, which may influence the effectiveness of mask ventilation.

We conducted this study to investigate three methods of RFM feedback, comparing them to a no-feedback control, assessing ventilation parameters and user interpretation. Three distinct visual feedback phases were tested, using standardised equipment and ventilation settings on a modified manikin.

## Methods

This simulation-based, crossover randomised study was conducted at the National Maternity Hospital (NMH), Dublin, Ireland. The protocol was approved by the local Research Ethics Committee (EC39.2022), and prospective written informed consent was obtained from all participants. The unit of analysis was the individual participant's interaction with different RFM feedback and its impact on leak reduction. The study follows reporting guidelines for health care simulation research.^10^

### Participants

Eligible participants included healthcare professionals of all experience levels involved in neonatal care at NMH, including neonatal nurses and paediatric or neonatal doctors. No prior experience with RFMs or simulation was required. Exclusion applied to individuals unable to provide written informed consent.

### Equipment

We modified a Laerdal Resusci Baby manikin (Dräger, Lubeck, Germany) to create a leak-free system.^11^ Ventilation was provided with a T-piece resuscitator (Neopuff) and a 60 mm round face mask (Fisher & Paykel, New Zealand), with gas flow at 10 L/min, peak inflating pressure (PIP) 20 cmH_2_O, and positive end expiratory pressure (PEEP) 5 cmH_2_O.

Two devices were used: Monivent NeoTraining (Göteborg, Sweden) and Respironics NM3 (Philips, USA). Both use differential pressure pneumotachometers between the resuscitation device and mask to measure airflow and pressure, calculating tidal volume (Vt). The Monivent RFM wirelessly transmits data to an iPad (Apple Inc.,) providing feedback on leak (%), expiratory tidal volume (Vt_e_) (ml/kg), ventilation rate (per minute), PIP and PEEP (cmH_2_O). Vt_e_ is displayed numerically and as a colour-coded cylinder: red (low), green (target), and orange (high). This was the “Coloured graphic” display. An LED on the sensor module also changes colour based on Vt_e_. The Monivent was set for a 3kg simulated weight and target Vt_e_ of 4–8 ml/kg.^12^ The Respironics RFM provides continuous numerical feedback on PIP and PEEP (cmH_2_O), Vt_e_ (millilitres) and ventilation rate (per minute). It also displays flow (litres/minute) and pressure curves (cmH_2_O), referred to as the “Flow curves” display.

### Study procedure

**Fig. 1:**
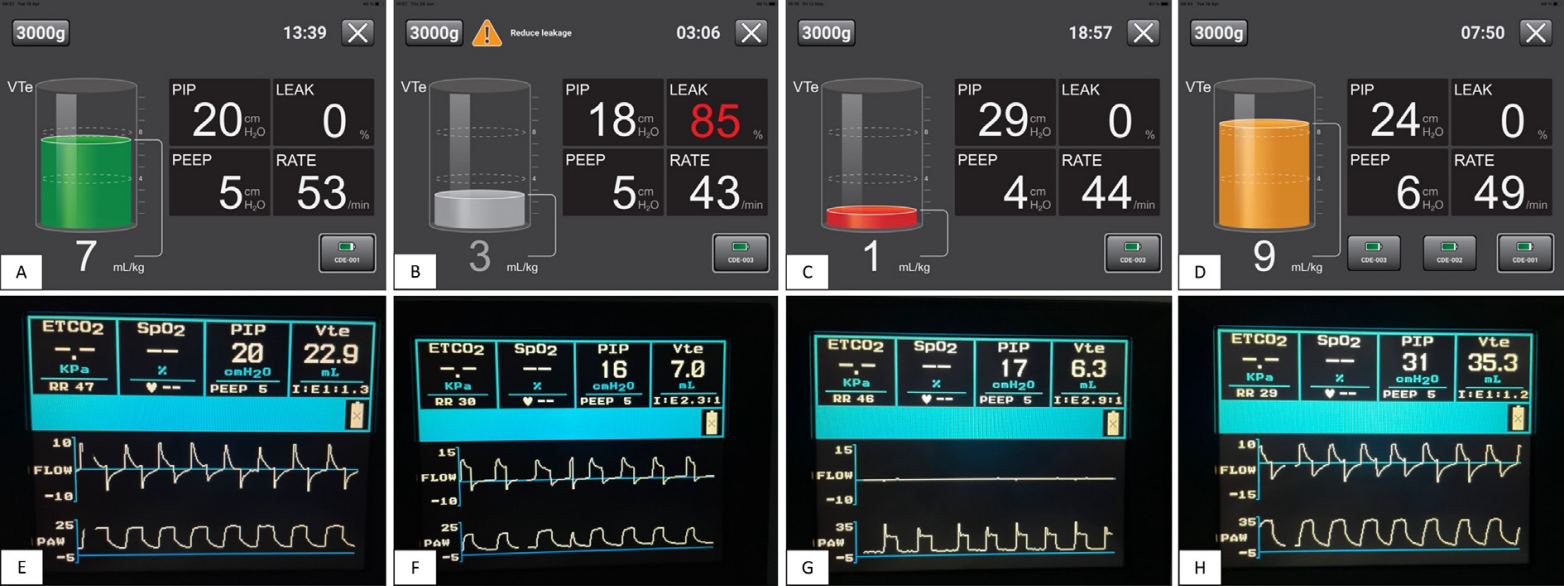
Visual displays from the “Coloured graphic” monitor (A – D) and “Flow curves” monitor (E – H) during optimal ventilation (A & E), under-ventilation due to high leak (B & F) and obstruction (C & G), and over-ventilation (D & H).

Each participant received standardised training on RFM interpretation using images of four scenarios from both devices: optimal ventilation, under-ventilation (high leak/obstruction), and over-ventilation (Fig. 1). The training focused on interpreting display data and correcting suboptimal inflations.

Participants then performed positive pressure ventilation (PPV) on a manikin in three phases with different RFM feedback, plus a control phase with no feedback. Training and PPV phases were conducted consecutively in the same clinical room. In the “Coloured graphic” phase, both the Monivent monitor and sensor light were visible. During the “Light only” phase, the Monivent monitor was concealed, and only the sensor light was visible. In the “Flow curves” phase, the Respironics monitor was visible. In the control phase, both the Monivent monitor and sensor light were concealed, with the light covered by opaque tape. In each phase, participants performed PPV for 30 s and aimed for leak <30%. 3, 4, 7, 12, 13 Both RFMs provided visual feedback and measured ventilation outcomes.

### Randomisation

Participants were randomly assigned to one of two sequences: (A) Coloured graphic *–* Light only *–* Control *–* Flow curves, or (B) Flow curves *–* Control *–* Coloured graphic *–* Light only. We generated the group assignment schedule in blocks of 4 using a random numbers table. Assignments were written on cards and placed in sequentially numbered, sealed, opaque envelopes.

### RFM interpretation

After the practical portion of the study, participants completed an assessment on RFM interpretation consisting of eight, two-part questions. Four scenarios from each RFM were presented: optimal ventilation, under-ventilation due to high leak and airway obstruction, and over-ventilation (Fig. 1). For each scenario, participants were required to identify the situation and select corrective action(s).

### Outcomes

The primary outcome was the median difference in face mask leak (%) between the control and the three intervention phases: “Coloured graphic”, “Light only” and “Flow Curves”, with data clustered by participant. Secondary outcomes included the mean differences in PIP, PEEP, Vt_e_ and ventilation rate between the control and interventions. Identification of optimal, under, and over-ventilation based on the “Coloured graphic” and “Flow curves” displays, along with the assessment of corrective actions taken in response to these scenarios, were also evaluated.

### Sample size and statistical analysis

We assumed a mask leak of 50% (SD 30%) in the control phase, based on previous studies.^2^ At the time of study design, the “Coloured graphic” monitor was novel and we lacked sufficient data to estimate the effect size. Therefore, an a priori sample size calculation was not performed, and we aimed to recruit a convenience sample of 50 participants. Data were analysed using Stata software (StataCorp, V.18, Texas, USA). Continuous data are presented as mean (SD) when normally distributed and median (IQR) when skewed. Mixed-effects quantile regression analysis was performed to assess effect sizes on non-parametric data, with a random intercept for participant. Outcome assessors were blinded to group allocation.

## Results

Fifty-six participants were randomly assigned over seven days in July 2023. The RFM data from five participants was corrupted, leaving 51 for analysis (Table 1): 25 (49%) sequence A, 26 (51%) sequence B.

**Table 1:** Participant demographics.

Characteristic	Detail	Participants n = 51
Professional role	Neonatal nurse	26 (51)
	Junior paediatric trainee a	10 (20)
	Senior paediatric trainee b	13 (25)
	Neonatology trainee	2 (4)
Previous RFM experience	Respironics NM3	0 (0)
	Monivent NeoTraining	4 (8)
	Florian	0 (0)
	Other	0 (0)
< 2 years since completion of newborn resuscitation training		49 (96)
Experience in newborn care (years), Median (IQR)		5 (2––13)

Data are number (%) unless otherwise stated.

a <6 months tertiary neonatal experience.

b >6 months tertiary neonatal experience.

### RFM data

**Fig. 2:**
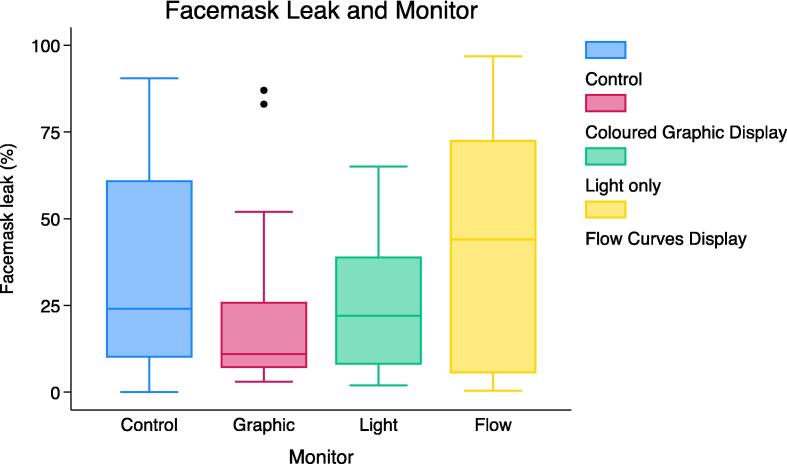
Leak (%) distribution for various RFM feedback displays and a no feedback condition. The horizontal line inside each box represents the median, the top and bottom of the box represent the IQR and the whiskers represent the adjacent values (most extreme values within 1.5 IQR of the nearest quartile). Circles plotted beyond the whiskers represent outliers.

The median (IQR) mask leak (%) was significantly lower in the “Coloured graphic” phase (11% [7%–26%]) compared to the control (24% [10%–61%]); median difference (MD): −13 [95% CI: −26 to −2]) (Fig. 2, Table 2). No significant differences were observed in the “Light only” phase (22% [8%–39%]); MD: −10 [95% CI: −25 to 5]) or the “Flow curves” phase (44% [6%–73%]; MD: 8 [95% CI: −2 to 18]) compared to the control. Secondary outcomes are detailed in Table 2.

**Table 2:** Outcomes across all phases; median differences compared with control.

	Control n = 51	Coloured graphic n = 51	Light only n = 51	Flow curves n = 51
**Primary Outcome**				
Leak (%) a MD (95% CI)	24 (10, 61)	11 (7, 26) −13 (−26, −2)	22 (8, 39) −10 (−25, 5)	44 (6, 73) 8 (−2, 18)
**Secondary Outcomes**				
Vt_e_ (ml) MD (95% CI)	16.0 (4.7)	18.2 (3.0) 1.8 (0.1, 3.5)	17.8 (2.4) 1.1 (−0.3, 2.5)	15.4 (4.9) −1.0 (−2.6, 0.6)
PEEP (cmH_2_0) MD (95% CI)	4.5 (0.8)	4.7 (0.7) 0.2 (0.01, 0.4)	4.7 (0.8) 0.1 (−0.1, 0.3)	4.8 (0.6) (−0.04, 0.6)
PIP (cmH_2_0) MD (95% CI)	19.1 (0.8)	19.4 (0.7) 0.3 (0.1, 0.5)	19.3 (0.6) 0.1 (−0.1, 0.3)	19.1 (0.8) 0.1 (−0.2, 0.3)
Rate (breaths/min) MD (95% CI)	46 (10)	47 (10) 0.5 (−1.8, 2.8)	47 (10) 0.6 (−1.0, 2.1)	42 (9) −3.8 (−6.4, −1.2)

Data are mean (SD) unless otherwise stated.

Abbreviations: MD, median difference. CI, confidence interval. Vt_e_, expired tidal volume. PEEP, Positive end expiratory pressure. PIP, Peak inspiratory pressure.

a Median (IQR).

### RFM interpretation assessment

More participants correctly identified the visual display from the “Coloured graphic” monitor compared to the “Flow curves” display for optimal ventilation (94% vs 78%), high leak (90% vs 73%), and over-ventilation (90% vs 75%) (Table 3). However, despite accurate identification, participants often failed to choose appropriate corrective actions. For optimal ventilation, more participants selected the “no change” action when interpreting the “Coloured graphic” display compared to the “Flow curves” (90% vs 75%,). For both devices, participants rarely selected the correct responses for addressing high leak (26% vs 24%) and obstruction (16% vs 14%) (Table 3).

**Table 3:** RFM interpretation assessment.

	Coloured graphic n = 51	Flow curves n = 51
***Correct interpretation of visual display***		
1. Optimal ventilation	48 (94)	40 (78)
2. Under-ventilation (High leak)	46 (90)	37 (73)
3. Under-ventilation (Obstruction)	44 (86)	38 (75)
4. Over-ventilation	46 (90)	38 (75)
***Correctly chosen action(s)***		
1. No change	46 (90)	38 (75)
2. Readjust mask & Reposition airway	13 (26)	12 (24)
3. Reposition airway & Readjust mask & Suction	8 (16)	7 (14)
4. Adjust PIP	40 (78)	35 (69)

Data are n(%).

## Discussion

In this simulated crossover study, participants were exposed to three feedback displays during mask ventilation, as well as a period without feedback. The lowest leak occurred when participants used the “Coloured graphic” monitor with its sensor light. The “Flow curves” display resulted in lower inflation quality, likely due to its complexity, which may have distracted participants. Despite providing more detailed information, the “Flow curves” display may have been less intuitive, possibly leading to cognitive overload and poorer performance compared to the control. Previous studies on RFMs in clinical resuscitations have shown inconsistent improvements in outcomes,^4^, ^5^, ^6^, ^7^ possibly due to challenges in interpreting feedback,^9^ as many devices may not have been designed with ease of use in mind. Newer generation devices, such as the “Coloured graphic” monitor with its simplified display, could help address this issue.

The interpretation assessment provided valuable insights. Although more participants accurately interpreted the “Coloured graphic” display, this did not lead to a greater selection of appropriate corrective actions. Despite recognising issues, only a minority chose the appropriate actions to address high leak and obstruction, highlighting the need for more comprehensive training on how to correct suboptimal inflations using RFM feedback.

## Strength and limitations

To our knowledge, this is the first simulation study to investigate how different visual feedback affects the quality of inflations during mask ventilation. While including both nursing and medical professionals strengthens the study, the use of a manikin model may limit its applicability. This study was conducted at a single centre with a small convenience sample, which limits generalisability. We used two different RFMs to measure ventilation parameters. Potential systematic differences in measurement accuracy and data processing between devices may have influenced the results. The study design was pragmatic, however the crossover of interventions may introduce bias from learning effects and similarities between phases limited statistical analysis. Potential confounders included clinician experience and the order of feedback phases. Additionally, we did not reassess participants’ retention of interpretation skills, which is known to deteriorate over time. 14, 15, 16

## Conclusion

Visual feedback from a “Coloured graphic” RFM during simulated neonatal face mask ventilation led to a significant reduction in mask leak.

## CRediT authorship contribution statement

**C.M. Ní Chathasaigh:** Writing – original draft, Methodology, Investigation, Formal analysis, Data curation, Conceptualization. **A.E. Curley:** Writing – review & editing, Supervision, Methodology, Conceptualization. **E. O Currain:** Writing – review & editing, Supervision, Methodology, Formal analysis, Conceptualization.

## Funding

The National Women and Infants Health Programme provided PhD funding for Dr Ní Chathasaigh during the conduct of this study (no award/grant number). The funder had no role in study design, data collection, data analysis, data interpretation, or writing of the manuscript.

## Declaration of competing interest

The authors declare that they have no known competing financial interests or personal relationships that could have appeared to influence the work reported in this paper.
