# Supplementary material for: Comparison of two respiratory function monitors for newborn mask ventilation: A randomised crossover study using simulation
Source: Resusc Plus. 2025 Mar 20;23:100937. doi: 10.1016/j.resplu.2025.100937 (PMC12018568; doi:10.1016/j.resplu.2025.100937)
Supplement: Supplementary Data 1 [file mmc1.docx]

| **CONSORT Item Number** | **CONSORT Description** | **Extension for Simulation-Based Research (SBR)** | **Page** |
| --- | --- | --- | --- |
| **Title and Abstract** |  |  |  |
| 1. Title | a. Identification as a randomized trial in the title | In the abstract or key terms, the MESH or searchable keyword term must include the word "simulation" or "simulated." | Title page |
|  | b. Structured summary of trial design, methods, results, and conclusions | Clarify whether simulation is the subject of research or an investigational method for research. | Title page |
| **Introduction** |  |  |  |
| 2. Background | a. Scientific background and explanation of rationale | Describe the theoretical and/or conceptual rationale for the design of each intervention. | 3 |
|  | b. Specific objectives or hypotheses | Clearly describe all simulation-specific exposures, potential confounders, and effect modifiers. | 3 |
| **Methods** |  |  |  |
| 3. Trial Design | a. Description of trial design (e.g., parallel, factorial), including allocation ratio | Clearly indicate the unit of analysis (e.g., individual, team, system), identify repeated measures on subjects, and describe how these issues were addressed. | 3 |
|  | b. Important changes to methods after trial commencement (e.g., eligibility criteria), with reasons |  | n/a |
| 4. Participants | a. Eligibility criteria for participants | Include participants' previous experience with simulation and other relevant features. | 4 |
|  | b. Settings and locations where data were collected |  | 5 |
| 5. Interventions | a. The interventions for each group, with sufficient details to allow replication, including how and when they were administered | In describing the methods of assessment, include the setting, instrument, simulator type, timing in relation to the intervention, and methods to enhance measurement quality. | 4-5 |
| **Outcomes** |  |  |  |
| 6. Outcome Measures | a. Completely defined prespecified primary and secondary outcome measures, including how and when they were assessed | Provide evidence to support the validity and reliability of assessment tools in this context (if available). | 6 |
|  | b. Any changes to trial outcomes after the trial commenced, with reasons |  | n/a |
| **Sample Size/Study Size** |  |  |  |
| 7. Sample Size | a. How sample size was determined |  | 6 |
|  | b. When applicable, explanation of any interim analyses and stopping guidelines |  | n/a |
| **Randomization** |  |  |  |
| 8. Sequence Generation | a. Method used to generate the random allocation sequence |  | 5 |
|  | b. Type of randomization and details of any restriction (e.g., blocking and block size) |  | 5 |
| 9. Allocation Concealment Mechanism | a. Mechanism used to implement the random allocation sequence (e.g., sequentially numbered containers), describing steps taken to conceal the sequence until interventions were assigned |  | 5 |
| 10. Implementation | a. Who generated the random allocation sequence, who enrolled participants, and who assigned participants to interventions |  | 5 |
| **Blinding (Masking)** |  |  |  |
| 11. Blinding | a. If done, who was blinded after assignments to interventions (e.g., participants, care providers, those assessing outcomes) and how | Describe strategies to decrease the risk of bias when blinding is not possible. | 6 |
|  | b. If relevant, description of the similarity of interventions |  | n/a |
| **Statistical Methods** |  |  |  |
| 12. Statistical Methods | a. Statistical methods used to compare groups for primary and secondary outcomes | Clearly describe any statistical methods used for additional analyses, such as subgroup or adjusted analyses. | 6 |
|  | b. Methods for additional analyses (e.g., subgroup analyses and adjusted analyses) |  | n/a |
| **Results** |  |  |  |
| 13. Participant Flow | a. For each group, the numbers of participants who were randomly assigned, received intended treatment, and were analyzed for the primary outcome |  | 7 |
|  | b. For each group, losses and exclusions after randomization, together with reasons |  | n/a |
| 14. Recruitment | a. Dates defining the periods of recruitment and follow-up |  | 7 |
|  | b. Why the trial ended or was stopped |  | n/a |
| 15. Baseline Data | a. A table showing baseline demographic and clinical characteristics of each group |  | Table 1 |
| 16. Numbers analysed | For each group, number of participants (denominator) included in each analysis and whether analysis was by original assigned groups |  | 7 |
| 17. Outcomes and estimation | a. For each primary and secondary outcome, results for each group, and the estimated effect size and its precision (such as 95% confidence interval) b. For binary outcomes, presentation of both absolute and relative effect sizes is recommended | For assessments involving >1 rater, interrater reliability should be reported | 7  &  Table  2 |
| 18. Ancillary analyses | Results of any other analyses performed, including subgroup analyses and adjusted analyses, distinguishing prespecified from exploratory |  | n/a |
| 19. Adverse events | important harms or unintended effects in each group (for specific guidance, see CONSORT for harms) |  | n/a |
| 20. Discussion Limitations | Trial limitations, addressing sources of potential bias, imprecision, and, if relevant, multiplicity of analyses | Specifically discuss the limitations of SBR. | 8-9 |
| 21. Generalisability | Generalisability (external validity, applicability) of the trial findings | Describe generalisability of simulation-based outcomes to patient-based outcomes (if applicable) | 8 |
| 22. Interpretation | Interpretation consistent with results, balancing benefits and harms, and considering other relevant evidence |  | 8 |
| 23. Registration | Registration number and name of trial registry |  | n/a |
| 24. Protocol | Where the full trial protocol can be accessed, if available |  | n/a |
| 25. Funding | Sources of funding and other support (such as supply of drugs), role of funders | List simulator brand and if conflict of interest for intellectual property exists. | 10 |
